# Supplementary material for: Urban–rural differences in perception of trees described by parents bringing up children in Warsaw and Jedlińsk, Poland
Source: PeerJ. 2020 Apr 8;8:e8875. doi: 10.7717/peerj.8875 (PMC7150541; doi:10.7717/peerj.8875)
Supplement: Supplemental Information 1 [file peerj-08-8875-s001.docx]

Perception of trees by city and village residents

Dear Sir or Madam,

The aim of the study is to assess the perception of trees and their importance for the city and village residents. The study will serve as part of a scientific article with a similar title.

The survey consists of questions about the benefits of trees, tree perception and defects and threats from trees, and supplementary questions. At the end of the questionnaire there is a short record containing questions concerning respondents’ profile. The survey is anonymous. Please, mark your choices with a cross.

Thank you

Do you agree with the following statements regarding mature trees?

|  | 1  Definitely not | 2  No | 3  Rather no | 4  Do not know | 5  Rather yes | 6  Yes | 7  Absolutely |
| --- | --- | --- | --- | --- | --- | --- | --- |
| Make urbanized environment* more pleasant to live, work and spend leisure time |  |  |  |  |  |  |  |
| Modify microclimate |  |  |  |  |  |  |  |
| Drop flowers and dirt the area around them |  |  |  |  |  |  |  |
| Significantly improve air quality |  |  |  |  |  |  |  |
| Reduce rate of storm water runoff |  |  |  |  |  |  |  |
| Drop branches and seeds and dirt the area around them |  |  |  |  |  |  |  |
| Contribute to road accidents |  |  |  |  |  |  |  |
| Improve physical health |  |  |  |  |  |  |  |
| Cause danger on playgrounds (and therefore should be removed) |  |  |  |  |  |  |  |
| Look beautiful in the autumn |  |  |  |  |  |  |  |
| Improve psychological health |  |  |  |  |  |  |  |
| Reduce noise |  |  |  |  |  |  |  |
| Are an escape from the hustle and bustle of the city, help people relax and calm down |  |  |  |  |  |  |  |
| Destroy road surface by roots |  |  |  |  |  |  |  |
| The presence of trees soothes nerves and stress |  |  |  |  |  |  |  |
| Limit the view from the windows of apartments and houses |  |  |  |  |  |  |  |
| Building stronger sense of community |  |  |  |  |  |  |  |
| Limit the access of light |  |  |  |  |  |  |  |
| Are a threat to human security due to fragile branches |  |  |  |  |  |  |  |
| Looking beautiful while blooming |  |  |  |  |  |  |  |
| Reduce crime |  |  |  |  |  |  |  |

Key: *Urbanized environment meaning public spaces or common spaces that have features of build-up areas that can be found in the city, in the suburbs or in the countryside.

Do you agree with the following statements regarding mature trees?

|  | 1  Definitely not | 2  No | 3  Rather no | 4  Do not know | 5  Rather yes | 6  Yes | 7  Absolutely |
| --- | --- | --- | --- | --- | --- | --- | --- |
| Bring the world of nature closer |  |  |  |  |  |  |  |
| Improve the aesthetics of the house and the environment |  |  |  |  |  |  |  |
| They are a cover for criminal activities |  |  |  |  |  |  |  |
| Provide shade on buildings sunny days |  |  |  |  |  |  |  |
| Control wind |  |  |  |  |  |  |  |
| In areas with trees, drivers are more careful and reduce speed |  |  |  |  |  |  |  |
| They attract unwanted insects |  |  |  |  |  |  |  |
| Extend the life of the surface |  |  |  |  |  |  |  |
| Provide shade on buildings sunny days |  |  |  |  |  |  |  |
| Help to save energy |  |  |  |  |  |  |  |
| Trees increase the value of the property on which they are located |  |  |  |  |  |  |  |
| Litter the area around through falling leaves |  |  |  |  |  |  |  |
| Cause allergies |  |  |  |  |  |  |  |
| They are a desirable habitat for many important life around me  organisms |  |  |  |  |  |  |  |
| Provide privacy |  |  |  |  |  |  |  |
| Costs of maintaining mature trees overwhelm their benefits |  |  |  |  |  |  |  |
| Drip sap or sticky residue on parked cars |  |  |  |  |  |  |  |
| Old, damaged trees are visually unattractive |  |  |  |  |  |  |  |
| It pays to plant trees (e.g. profit from energy saving) |  |  |  |  |  |  |  |
| Cover unpleasant views |  |  |  |  |  |  |  |
| Have a positive impact on children's development |  |  |  |  |  |  |  |

Are you trying to protect children from trees (prohibiting climbing trees, touching rotten parts, playing with leaves, branches? Other (please enter)

……….................................................................................................................. )

| Definitely not | No | Rather no | Do not know | Rather yes | Yes | Absolutely |
| --- | --- | --- | --- | --- | --- | --- |

RESPONDENTS’ PROFILE

1. Gender

| Female | Male |
| --- | --- |

1. Age

| Under 30 years | 30-45 years | 46-60 years | Over 60 years |
| --- | --- | --- | --- |

1. Education

| Primary school | High school | Higher education |
| --- | --- | --- |

1. Place of origin

| Village | City |
| --- | --- |

1. Place of residence

| Village | City |
| --- | --- |
